# Fast and Conspicuous? Quantifying Salience With the Theory of Visual Attention

**DOI:** 10.5709/acp-0184-1

**Published:** 2016-03-31

**Authors:** Alexander Krüger, Jan Tünnermann, Ingrid Scharlau

**Affiliations:** Faculty of Arts and Humanities, Paderborn University

**Keywords:** salience, visual attention, Bayesian inference, theory of visual attention, computational modeling

## Abstract

Particular differences between an object and its surrounding cause salience, guide attention, and improve performance in various tasks. While much research has been dedicated to identifying which feature dimensions contribute to salience, much less regard has been paid to the quantitative strength of the salience caused by feature differences. Only a few studies systematically related salience effects to a common salience measure, and they are partly outdated in the light of new findings on the time course of salience effects. We propose Bundesen’s Theory of Visual Attention (TVA) as a theoretical basis for measuring salience and introduce an empirical and modeling approach to link this theory to data retrieved from temporal-order judgments. With this procedure, TVA becomes applicable to a broad range of salience-related stimulus material. Three experiments with orientation pop-out displays demonstrate the feasibility of the method. A 4th experiment substantiates its applicability to the luminance dimension.

## Introduction

As early as 1890, William James (1890, p.416) described a kind of attention caused by “an instinctive stimulus, a perception which, by reason of its nature rather than its mere force, appeals to one of our normal congenital impulses”.

Though over a century old and in an uncommon wording, the quote expresses the idea that some objects trigger basic attentional mechanisms that all humans share. These mechanisms are feature-specific instead of being based on sensory strength. This description fits the current idea of stimulus-driven or *bottom-up* attention. For both James’ description and the modern perspective, however, there remains the question which features attract such attention. Among James’ rather uncommon examples are strange things, moving things, bright things, and metallic things. From today’s knowledge, we would argue that it is not simply the properties, but the context in which the object occurs which are of great importance. This relation is captured by the term *salience* (among others) which describes a local feature difference that attracts attention. Thus, a bright stimulus among other bright stimuli would not attract much attention, and neither would an object moving in the same direction and with the same speed as other moving objects.

James’ (1890) initial question which features are essential for guiding attention has been extensively studied within visual attention research (for a summary see Wolfe & Horowitz, 2004). However, much less research has addressed the strength of salience dimensions and their quantitative influence on attention, which is the focus of the present article. If you want to be seen, would it be better to be moving, or to be bright—or even metallic?

There are several, mostly model-based approaches to answer this question.

Early visual processing is based on the receptive fields of neurons tuned to particular features (e.g., Hubel & Wiesel, 1959, 1968), which are the source of bottom-up influences on perception and attention (for a review see Treue, 2003). The strength of these neurophysiological responses depends on the strength of the presented features (Zhang, Zhaoping, Zhou, & Fang, 2012). This strength and combinations of features of varying strength have predominantly been tackled using methods from engineering (e.g., Itti & Koch, 2001b; Zhao & Koch, 2013).

Computational modeling approaches allow to simulate retinotopic salience maps for natural input images (for a review see Frintrop, Rome, & Christensen, 2010). Different mathematical strategies have been explored to compute a salience value for every location in the image. Because of the difficulties of solving these problems algorithmically, machine learning techniques have been employed (Itti & Koch, 2001b; Zhao & Koch, 2013). Although such approaches may be applied in computer vision, it is unclear if they correspond to salience in human attention. For instance, many computational models such as that by Itti and Koch (2001a) predict that a higher luminance contrast attracts more attention. Einhäuser and König (2003) experimentally manipulated the luminance contrast of images. The participants in their study had to carefully study natural and modified natural images. The correlation of luminance contrast and fixation probability, however, failed to confirm the model prediction.

The neurophysiological salience model by Li (2002) makes quantitative predictions about human performance in salience related tasks. Li assumes that the strength of salience is represented implicitly by the firing rate of retinotopic neurons in V1 that encode specific features or combinations of features. This model accounts qualitatively for a wide range of empirical findings like search asymmetries in visual search (e.g., Li, 1999). It simulates the neurophysiological processing of the visual information by a complex recurrent artificial neuronal network (Li, 2001). The firing rate of these artificial neurons can hence be regarded as a quantitative prediction. However, the model cannot yet account quantitatively for experimental data.

Another model focusing on salience-related human performance is the fourth version of the Guided Search model by Wolfe (2007). In this model, salience is handled by a module for the bottom-up guidance of attention. This guidance is modeled by individual channels tuned to specific features (e.g., steep, shallow, left, and right for orientation). It contains a simple mathematical function for the contribution of each orientation channel. Salience itself is then computed by pairwise comparisons of these values for all visible objects. Wolfe states that the precise shape of the function that determines the contribution of a channel to overall salience is not critical for the qualitative performance of the model. This statement makes it questionable whether the model may provide good quantitative predictions on this level although it qualitatively accounts for a wide range of empirical findings on visual search. As Wolfe himself concedes, not all quantitative aspects of human behavior in terms of response times and errors can be successfully predicted. In conclusion, models do not yet provide a general explanation of the quantitative strength of salience.

Some attempts to establish a quantitative measure of salience are based on the analysis of behavioral data. Among the few studies in this line of research are those by Nothdurft (1993, 2000). He asked participants to compare the conspicuousness of two singletons that are unique elements embedded in a display of homogeneous background elements. Each stimulus whose salience was to be measured was presented with a stimulus that was salient due to a luminance difference. To measure the salience of a stimulus, the salience of the reference (luminance) stimulus was systematically increased. By this means, Nothdurft (2000) related the feature dimensions motion, orientation, luminance, and color to each other and also compared combinations of features from different dimensions. He quantified salience by relating a salient stimulus to the luminance difference that would create the same salience via approximation of psychometric functions and calculating what one might call the point of subjective equal salience. This approach comes close to a general and theoretically well-founded quantification. Unfortunately, the results are difficult to replicate. While we could replicate Nothdurft’s findings using orientation and luminance, we also found that many participants showed no regular psychometric functions but rather a behaviour strongly influenced by guessing (unpublished pilot study). Similar difficulties were reported by Koene and Zhaoping (2007).

Starting from this need for a better behavioral method to quantify salience, Huang and Pashler (2005) came up with a search task for the biggest and brightest square in a display of several objects. The location of a small probe on its left or right side had to be reported to verify that the target was found. The dependent variable was the response time for a correct report. The display was randomly filled with other distractor squares. Salience was measured in these trials by introducing a salient key distractor. Its salience was quantified by examining the effect of the feature differences on response times. Via this quantification, Huang and Pashler related luminance and size to each other.

An additional aspect impeding the measurement of salience is its time course. Regarding the time course, several different ideas were discussed (e.g., Egeth & Yantis, 1997), with two types of temporal dynamics being especially important for the study of salience. (1) Salience-based *progression of attention* (e.g., Koch & Ullman, 1985) describes the shift of attention from the most salient spot in an image to the second most salient spot and so forth. (2) *Time course of salience* describes how the strength of salience effects varies over time. Salience effects increase from display onset to 100 or 150 ms (e.g., Couffe, Mizzi, & Michael, 2016; Kean & Lambert, 2003) and decay after approximately 300 ms. Evidence for this time course—which resembles the time course of attention (Olivers, 2007)—comes from a variety of different paradigms: probe detection (Dombrowe, Olivers, & Donk, 2010; Donk & Soesman, 2010), TOJs (Donk & Soesman, 2011), saccadic selection (Donk & van Zoest, 2008), and saccadic trajectories (van Zoest, Donk, & Van der Stigchel, 2012). This research implies that it is crucial to measure salience at specific points in time (a condition not met by Huang & Pashler, 2005).

The approaches discussed above consider or measure performance as an indicator of attention. They spend less effort on the quantification of salience itself. An approach that might provide such a quantification is Bundesen’s Theory of Visual Attention (TVA; Bundesen, 1998). It comprises a psychologically inspired, general formal explanation of visual attention and selection processes and allows to infer attentional weights for specific objects in a display. The attentional weight determines if an object is encoded in visual short-term memory (VSTM)—and if so, how quickly it is encoded—that is, its processing speed. These parameters can possibly be used as a general quantification of salience in the sense that the strength of salience is the attentional weight of an object.

Although promising on an abstract level, TVA has only rarely been used to investigate salience (e.g., Nordfang, Dyrholm, & Bundesen, 2013). A possible reason is that in the item-report paradigms commonly used with TVA, the potential stimulus material is restricted to highly overlearned categories like digits and letters. The experimental paradigm requires a categorization because probabilities of stimulus categorizations are estimated. Hence, TVA is not directly applicable to salience research.

Recently, however, Tünnermann, Petersen, and Scharlau (2015) paved the way for such an application. Originally, they investigated whether the relatively faster perception of an attended stimulus in a pair is caused by speeded processing of this attended stimulus or decelerated processing of its unattended counterpart. Along with TVA-based item report, participants judged the temporal order (temporal-order judgment; TOJ) in which the stimuli appeared. Tünnermann et al. found that the attentional benefit originates from a combination of speeding up the attended and slowing down the unattended stimulus. This conclusion is based on a conventional TVA analysis. In the Discussion, however, they sketched a new approach. They suggested that data from TOJ might be directly modeled by TVA to obtain TVA’s attention parameters. At first sight, this might not seem ground-breaking, but the proposed method offers applying TVA-based analysis to any kind of stimulus. The aim of the present paper is to test the feasibility of this approach.

In a nutshell—details will be explained below in two sections on TVA and modeling of TOJ data—the method consists of having observers judge the temporal order of two arbitrary visual stimuli. The interval between the stimuli is varied over trials. Application of TVA to the observers’ judgments allows computing of processing speed, attentional weights, and overall attentional processing capacity. By manipulating the features of the stimulus, this method allows us to quantify salience in the form of these parameters. This approach can provide a theoretically well-founded, general quantification of salience.

### The Theory of Visual Attention (TVA)

The present section provides a short summary of the relevant parts of TVA as a formal theory. Key terms for the modeling as well as the experiments are introduced, most importantly *attentional weight* and *processing capacity*. The section can, however, not provide a full introduction to TVA, for which we refer the interested reader to sources such as those by Bundesen (1998) and Bundesen, Habekost, and Kyllingsbæk (2005).

TVA was introduced as a unified theory of visual recognition and attentional selection. The theory achieves this by mathematically formalizing the processes associated with the processing of visual objects from presentation towards encoding in VSTM. This processing is described as a race for representation in one of the limited slots in VSTM. Stimuli race independently and in parallel. The race is influenced by many factors. Among them are the total number of elements competing for representation, the distribution of attention across the stimuli, and the categories to which the stimuli potentially belong.

In order to explain the formalization of this process, we proceed backwards from the arrival in VSTM to the appearance of the stimuli.

TVA assumes that the arrival times of stimuli in VSTM are exponentially distributed. Although the theory is fleshed out for multiple stimuli, the present approach is a simpler case: In the derivation proposed by Tünnermann et al. (2015) on the basis of TOJs, only two targets are encoded. Thus, the VSTM limitation can be ignored, which simplifies formalization. Back to the event of encoding an object to VSTM, the probability of an object *x* to be encoded before time *t* can then be expressed as the probability density function:


(1) $$F\left(t\right)=\left\{ \begin{array}{c} 1-e^{-v_{x}\left(t-t_{0}\right)}\mathrm{if}t>t_{0}\\ 0,\mathrm{else} \end{array}\right.$$

The two cases that are distinguished in the equation emerge from the assumption that there is a maximal ineffective exposure duration *t*_0_. This is the interval—that is still too short to provide enough sensory evidence for the race to start at all. If *t* ≤ *t*_0_, there is no chance that the processing of *x* finishes, whereas for *t* > *t*_0_ there is a chance that processing has been completed. This probability depends on the exposure duration and the processing rate υ_x_. This rate’s unit corresponds to categorizations per second, and it is composed of:


(2) $$v_{x}=\sum_{i\in R}v\left(x,i\right)$$

The equation is based on the idea that different categorizations are possible for object *x*. The set *R* represents this set of categories and the processing rate υ(*x*,*i*) with expressing the speed of the particular categorization that *x* belongs to category _i_. This *i* can, for example, refer to the property of having a particular color or a certain orientation.

Descending deeper into the formalization, the processing rate is defined as:


(3) $$v\left(x,i\right)=\eta \left(x,i\right)\beta_{i}\frac{w_{x}}{\sum_{z\in S}w_{z}}$$

This equation introduces three important factors that are η(*x*,*i*), the strength of the sensory evidence that *x* belongs to category *i*, β_i_, a decision bias for category *i*, and the relative attentional weight for *x* given by its own weight ω_x_ divided by the weights for all objects in the visual field. All objects in the visual field are contained in the set *S*. The weights are defined by the weight equation:


(4) $$w_{x}=\sum_{j\in R}\eta \left(x,j\right)\pi_{j}$$

which again includes the sensory evidence for *x* as η(*x*,*j*) and a new variable Π_j_, which is a selection bias for category *j*, the pertinence value. These are summed over the set of all categories *R*.

The present approach concentrates on the parameters attentional weight ω, processing speed υ, and overall processing capacity *C*. The processing speed describes how quickly a representation in VSTM is built up. The sum of all the processing speed available is the processing capacity. The attentional weight corresponds to the relative advantage of a stimulus and expresses how much attention is allocated to this object in comparison to the others. (The biases Π and β are both held constant in the context of the present experiments and are hence not estimated.)

Based on this admittedly swift introduction of the formalization the reader may deem TVA too cumbersome for dealing with comparably simple salience displays. This formalization, however, offers advantages. Firstly, TVA allows precise quantification and provides psychologically meaningful parameters, such as processing speed, which can be applied to a broad range of perceptual and attentional phenomena. Secondly, salience research can be related to other phenomena that have already been studied with TVA, such as, for example, feature-difference (bottom-up) and feature-relevance (top-down) interactions (Nordfang et al., 2013). Finally, because of its precise quantitative nature, the TVA framework can be used for generating quantitative hypotheses.

### Modeling TOJ Data by TVA

TVA was initially applied to multi-element displays of highly overlearned stimuli, such as letters or numbers from which all or several belonging to a certain category had to be reported. The stimuli have to be masked to derive the assumed performance. Both features—highly overlearned and maskable stimuli—have so far restricted the general applicability of TVA. As already mentioned, Tünnermann et al. (2015) discussed a TOJ model derived from TVA equations which renders TVA applicable to all kinds of visual stimuli and also does away with the necessity of masking. They did so by introducing a temporal-order task and relating the psychometric functions derived from this task mathematically to the distributions assumed by TVA. In the following section, we will explain briefly how TOJ data can be modeled with TVA. For more detail, we refer the reader to the original article.

In the TOJ paradigm, the temporal order of two onsets has to be judged. We call these two targets *T*_probe_ and *T*_reference_. In the experiments presented later, they will have different properties according to the experimental variable, but at present these names are just used to make them distinguishable. They appear with a variable interval between them. The dependent variable is the amount of judgments for *T*_probe_. If *T*_probe_ precedes *T*_reference_ with a large interval, judgments in favor of *T*_probe_ will be frequent. If the other stimulus leads, the proportion of judgments for *T*_probe_ will be low. If *T*_probe_ and *T*_reference_ are comparable, and the two stimuli are presented simultaneously, the participants’ performance should reach chance level.

However, subjective perception can deviate markedly from objective events. Such judgments can, for example, be systematically influenced by attention. If one of the stimuli is attended-to in advance, this stimulus will be perceived earlier. This phenomenon is called prior entry (Spence & Parise, 2010). In terms of the judgments, this effect becomes evident in an increased proportion of reporting the attended stimulus as being perceived first.

TOJ data can be fitted with psychometric functions. Possible mathematical descriptions of psychometric functions include the cumulative distribution of the normal distribution, logistic, Weibull, and Gumbel functions, of which the former two are most widely employed (for more formal descriptions and how to fit these functions see Kuss, Jäkel, & Wichmann, 2005; Wichmann & Hill, 2001a, 2001b). These functions have at least two parameters, the most important of which describe the center of the function and its slope. The center, at which both judgments are equally likely, is usually interpreted as the point of subjective simultaneity (though see Weiß & Scharlau, 2011). The slope is an indicator of discrimination performance. Importantly, it is a matter of debate which of the functions mentioned above should be used because none of them is particularly supported by theory. Hence, also the interpretation of the functions and their parameters is limited.

In contrast to psychometric functions, TVA offers parameters deeply rooted in psychological theory. As an additional advantage, they can also be interpreted readily. For instance, the parameter v corresponds to processing speed. Its unit is stimuli processed per second. This model carries more information than the point of subjective simultaneity and discrimination performance which measure only performance, not the processes that drive this performance.

**Figure 1.**
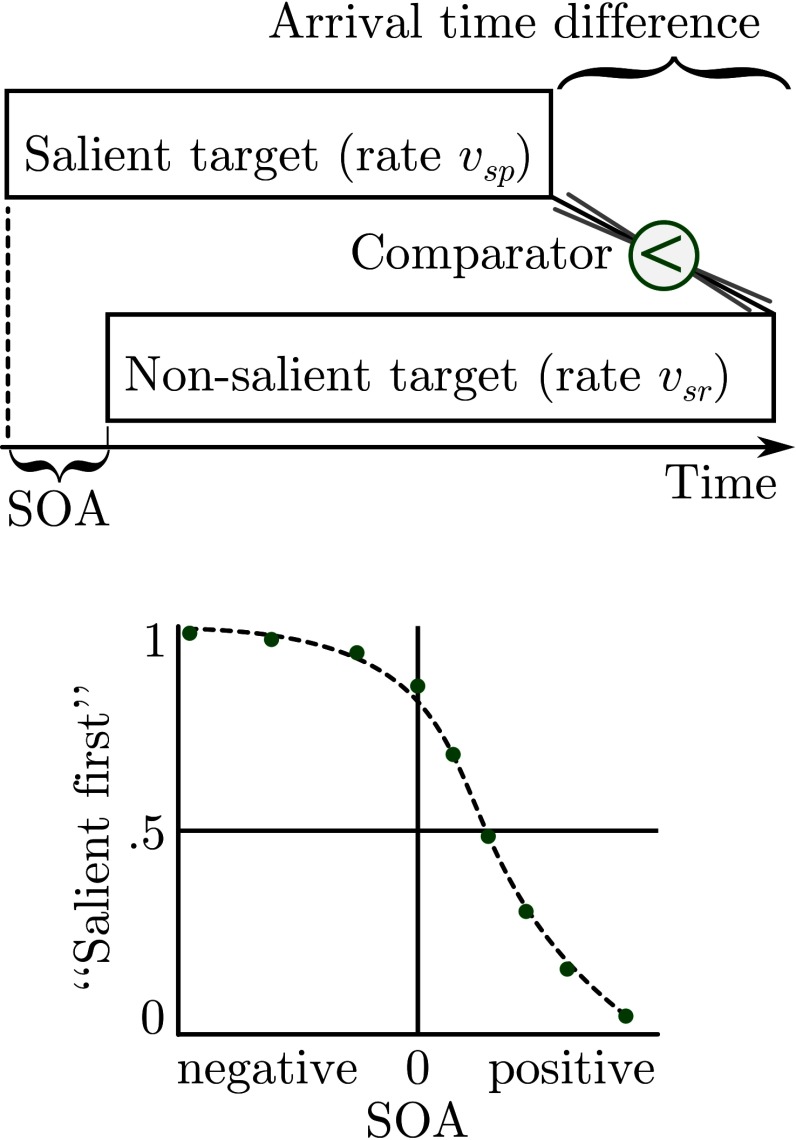
Cognitive model. The bars in the upper part represent the races to VSTM. Formally, these races depend on the processing rates. The rates υ_sp_ and υ_sr_ from the salience condition of the experiments are shown exemplarily. The proportion of “salient first” judgments depends on the comparison of both races. SOA = Stimulus Onset Asynchrony.

Each data point of a psychometric function is equivalent to the proportion of one event being encoded first. This connection is illustrated in Figure 1 for the judgment of a salient and a non-salient stimulus (the main conditions in the experiments reported below). Each of the points, sampled from the psychometric function, depends on the process depicted above the function: According to the TVA-based model, each of the two bars represents a race to VSTM. The results of these two races are compared which determines the participant’s judgment. Each race is influenced by the objective onset and its speed. The process is, however, still a stochastic process—that is, these variables do not fully determine the outcome.

As proposed by Tünnermann et al. (2015) the chance of onset *T*_probe_ being encoded first can be described with the parameters of TVA. It can be expressed by three parameters which include υ_p_ (the processing speed of *T*_probe_), υ_r_ (the processing speed of *T*_reference_), and Δ*t* which incorporates the SOA and the maximal ineffective exposure duration as Δ*t* = SOA + *t*_0_^p^ − *t*_0_^r^, where *t*_0_^p^ and *t*_0_^r^ are the maximal ineffective exposure durations for the two stimuli. They are assumed to be equal in the context of the present experiments.

In terms of these parameters, the probability of *T*_probe_ being encoded first can be expressed as:


(5) $$P_{p}\left(v_{p},v_{r},\Delta t\right)=1-e^{v_{p}|\Delta t|}+e^{v_{p}|\Delta t|}\left(\frac{v_{p}}{v_{p}+v_{r}}\right)\mathrm{for}\Delta t<0$$

where 1-*e*^-v_p_|Δ*t*|^ describes the probability that *T*_probe_ is fully encoded before *T*_reference_ starts the race to VSTM. The probability *e*^v_p_|Δ*t*|^ is the probability of the event that *T*_probe_ is not encoded before *T*_reference_ starts its race. Then the probability of encoding *T*_probe_ first is given by Luce’s choice axiom

$$\left(\frac{v_{p}}{v_{p}+v_{r}}\right)=\int_{0}^{\infty }v_{p}e^{-v_{p}t}\cdot e^{-v_{r}t}\mathrm{dt}$$.

For Δt ≥ 0 it holds that:


(6) $$P_{p}\left(v_{p},v_{r},\Delta t\right)=e^{v_{r}|\Delta t|}\left(\frac{v_{p}}{v_{p}+v_{r}}\right)\mathrm{for}\Delta t\geq 0$$

Here, analogously *e*^v_r_|Δ*t*|^, denotes the probability that *T*_reference_ is not encoded before *T*_probe_ starts its race. If this happens, the probability of *T*_probe_ being encoded first is given by Luce’s choice axiom.

**Figure 2.**
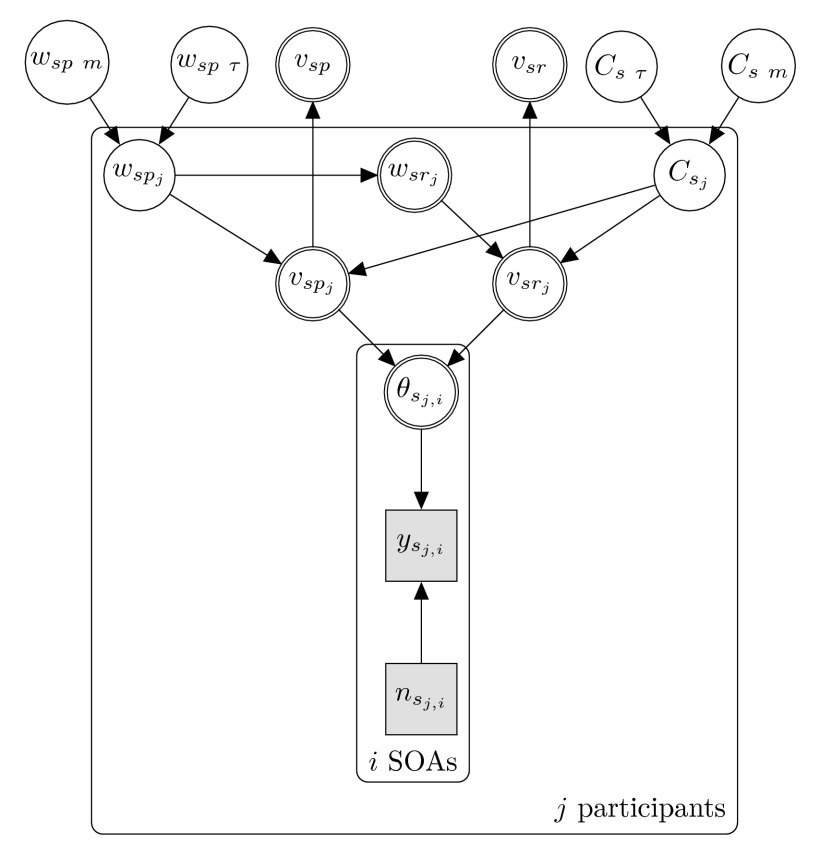
Hierarchical Bayesian graphical model of the data of the salience condition. The salience condition is indicated by the index *s* . The same model applies for the neutral condition *n*. The group level, the variables in the highest layer, estimate TVA parameters for a particular condition. This layer was compared to the neutral condition (see Table 1). SOA = Stimulus Onset Asynchrony.

To estimate the TVA parameters introduced in this section, a suitable statistical modeling is needed. We use Bayesian statistics for modeling and data analysis because Bayesian methods are particularly well-suited for inference under an assumed model (Little, 2006). We implemented a generative model based on the mathematical description of TVA, visualized in the hierarchical graphical Bayesian model of Figure 2. Table 1 shows how the variables (nodes) are formally defined. The graphical model describes the relation between the raw data and the TVA parameters on the group level. As an intermediate step, the TVA parameters are estimated per participant. The graphical model depicted in Figure 2 belongs to one group or condition in an experiment. Each further condition is modeled analogously. If there are at least two groups, their group parameters represented at the very top can be compared. On the group level, the mean of attentional weight is represented by node ω_sp m_. Because of technical reasons the variance of the estimated attentional weight is represented as a separate variable node ω_sp τ_. Similarly, the capacity mean and variance are represented by the upper two *C* nodes. Additionally, we can infer the group-level processing speed for both targets as represented by the upper υ nodes. However, they do not provide additional information because they depend on the weight and capacity, as indicated by the direction of the arrows. For further information on the exact nature of the Bayesian parameter estimation process, please refer to Appendix A.

**Table 1.** Variables of the Hierarchical Bayesian Graphical Model (See Figure 2)

Variable	Explanation
ω_npj_∼Normal(ω_npm_,ω_npτ_)	Attentinal weight (probe)
ω_nrj_=1-ω_npj_	Attentinal weight (reference)
ν_np_=mean(ν_npj_) j∈participants	Processing rate (probe)
ν_nr_=mean(ν_nrj_) j∈participants	Processing rate (reference)
C_nj_∼Normal(C_nm_,C_nτ_)	Processing capacity
ν_npj_=C_nj_·ω_npj_	Participant processing rate (probe)
ν_nrj_=C_nj_·ω_nrj_	Participant processing rate (reference)
θ_sj,i_←P_A_(ν_np_, ν_nrp_, SOA)	Probability of “Probe first”
y_nj,i_=Binominal(θ_nj,i_n_sj,i_)	Count “Probe first” response

The following four experiments test the viability of the proposed method in salience research. To this end, we combined TOJs with salience displays. In Experiment 1, the order of stimulus onsets had to be judged. This experiment was most similar to common TOJ experiments. In Experiment 2, stimulus offsets were judged, and the stimuli of Experiment 3 flickered for a short duration. We investigated whether salience increased processing speed and attentional weights. Finally, Experiment 4 was conducted to show the applicability to the luminance dimension as well as the sensitivity of the method.

## Experiment 1

Experiment 1 is based on the hypothesis that the onset of an orientation singleton achieves an increased attentional weight and is hence encoded to VSTM more quickly. It was carried out as a proof of concept to show that TVA can be successfully applied to salience research via the general TOJ method outlined by Tünnermann et al. (2015). To this end, it had to meet the requirements of both salience studies and TOJ research, requiring us to combine multi-element displays from salience research with temporally distributed targets in the most direct way possible.

**Figure 3.**
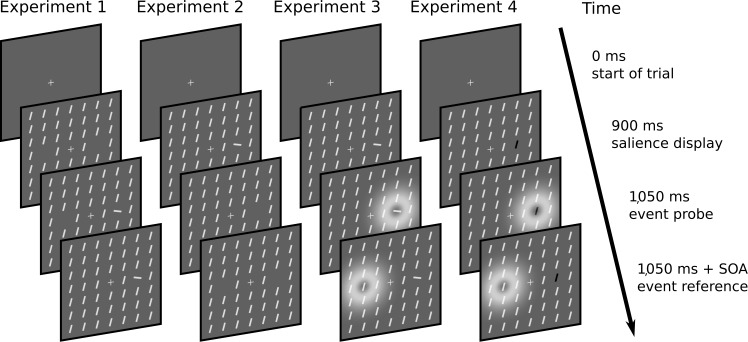
Visualization of the stimulus sequence of Experiment 1 to 4. Stimuli are identical to those of the experiments, but displays have been scaled for visibility. The salience display was shown 150 ms before the probe event. The event to be judged was the onset (Experiment 1), offset (Experiment 2), or flicker (Experiment 3 and 4; depicted as white coronae). Only the salience conditions are shown. These conditions comprise a salient probe stimulus. The neutral conditions of the experiments featured a non-salient probe stimulus equal to the reference stimulus. These conditions are not depicted. The arrow depicts the flow of time. SOA = Stimulus Onset Asynchrony.

The participants judged the temporal order in which two targets appeared in a display of 17 × 17 bars. A center section of these displays is exemplarily shown in Figure 3. The salience display consisting of homogeneous background stimuli was shown first. The targets appeared later. One of the targets could differ in orientation whereas the other one was always non-salient—that is, of the same orientation as the background elements.

This combination of multi-element displays and stimulus onsets is the direct way of checking the applicability of the method. Unfortunately, however, it is questionable whether target onsets allow salience effects to show up. Firstly, the blanks at the locations of the future targets may act as salient stimuli because they violate the background pattern (Li, 2002). Secondly, results on the temporal course of salience suggest that salience is used to gradually distribute attention over the display (Dombrowe et al., 2010): After a 30 ms delay, the salience effect is very small in comparison to its peak at 120 ms. Salience information thus might not be available initially. Finally, the onset information may be so strong that it masks any effects of salience. Because the present experiment serves as a proof of concept, this is no severe disadvantage. If the methodology works as expected, we will be able to precisely describe the reported temporal order with the help of the proposed model independent of whether an effect of salience is present on the group level. Following this proof of concept, Experiments 2 and 3 will look into effects of salience themselves.

### Method

#### Participants

A total of 20 students at Leuphana University of Lüneburg (5 male and 15 female; *M*_age_ = 23.9 years, range 20-33) participated in Experiment 1. Seven participants took part in an additional session and one participant in three sessions. Within Bayes methodology, such variation can be taken into account in the parameter estimation for the individual participants which improves precision. The higher precision on the individual level also affects the parameter estimation on the group level. All participants reported normal or corrected-to-normal visual acuity and received a payment of 8 Euro per hour.

#### Apparatus

The experiment was conducted in a dimly lit experimental booth.

A Windows 7 computer with a dedicated graphic card and an Iiyama Vision Master Pro512 22 inches (40.4 cm × 30.3 cm) CRT monitor was used for stimulus presentation. The refresh rate was set to 100 Hz, the resolution to 1,024 × 768 pixels with 32-bit colors. The vsync signal was used for timing the experiment. The experiment was programmed using PsychoPy (Peirce, 2007). The distance to the screen was 50 cm. Participants responded with the hand corresponding to the location that had to be reported. The control key on the bottom left and the enter key on the bottom right corner of the keyboard were used for responses.

#### Stimuli

Each trial started with a fixation cross in the center of the screen. After a delay of 900 ms, the participants saw a 17 × 17 array of bars. The array corresponded to 34.99° × 34.99° of visual angle. Bar length was 1.07° of visual angle and width 0.18°. The fixation cross occupied the middle of the array. The background color of the screen was set to gray, RGB (96, 96, 96) equivalent to 6.98 cd/m^2^ , while bars and fixation cross were white, RGB (224, 224, 224) equivalent to 66.2 cd/m^2^ . Each bar stimulus belonged to one of three logical categories which were not necessarily visually distinguishable. These categories are background elements, target *T*_reference_ and target *T*_probe_. While the background elements and *T*_reference_ were always homogeneously oriented, the orientation of *T*_probe_ varied between a 0° difference to the background in the neutral condition and the maximal orientation contrast of 90° in the salience condition. The orientation of the non-salient elements was chosen randomly for each trial. The targets were presented at fixed positions on the left and right of the fixation cross with an eccentricity of 8.24° of visual angle. Both positions were empty when the array was initially presented. *T*_probe_ was always presented 150 ms after the onset of the array of background elements. This duration was not jittered because salience effects decay over time as reported by, for example, Donk and van Zoest (2008), and the TOJ required a temporal window of -100 ms to +100 ms around this value. *T*_reference_ was shown with an SOA of -100, -80, -60, -40, -20, 0, 20, 40, 60, 80 and 1 ms, respectively. After a display duration of 300 ms, all bars vanished. The number of trials varied with the SOA because the variance is expected to increase towards the 0 SOA. Twenty-four trials were present for each of the -100, -80, 80, and 100 ms SOA, 32 trials for the -60, -40, 40 and 60 ms SOA, and 48 trials for the -20, 0, and 20 ms SOA. The participants had to respond via a keystroke with either the left ctrl or the right enter key. The side at which *T*_probe_ appeared was chosen randomly. The next trial started automatically with a delay of 1 s with a 100 ms jitter.

#### Procedure

Participants were instructed to fixate the cross in the center of the screen throughout each trial. Their task was to report which element occurred first, the left or the right one, and press the left or right key, respectively. There was no time pressure. The experiment started with a training phase of 40 trials that included feedback about errors. There was no feedback after the training. After 50 trials each, a break was initiated which was ended by a keypress. The experiment lasted approximately 45 min.

### Results

The judgments whether the left or right stimulus appeared first were converted into the judgment whether *T*_probe_ appeared first. Remember that *T*_probe_ is the stimulus that stands out from its surroundings in the salience condition.

**Figure 4.**
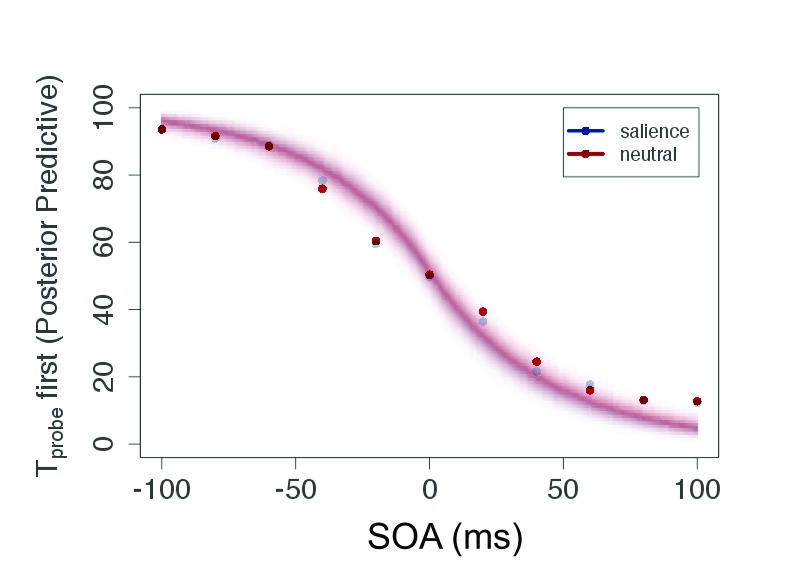
Plot of raw data (mean of judgment frequency per SOA over all participants) and posterior predictive for the salient and neutral condition of Experiment 1. This plot shows predicted data based on the estimated parameters. SOA = Stimulus Onset Asynchrony.

As can be seen in Figure 4, the participants generated typical sigmoid TOJ data. All individual data showed this pattern which allowed us to apply the model (see the section “Modeling TOJ data by TVA” for details).

Bayesian statistics yields a full probability distribution of the model parameters, a point estimate of the parameter, which is provided by the mode of the respective distribution, and an easily interpretable measure of the certainty with which the parameter was estimated. Broad probability distributions correspond to vague estimates. This information is expressed by the highest density interval (HDI) of the distribution, the interval on the *x*-axis in which 95% of the likely parameters lie.

**Figure 5.**
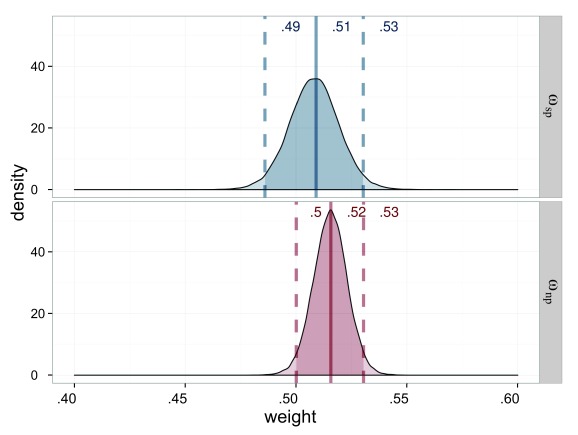
Estimated attentional weights (ω) for the probe stimuli of Experiment 1, salience condition (ω_sp_ = weight for the salient probe) in blue and neutral (ω_sp_ = weight for the neutral probe) in red. The weights for the reference stimuli are 1 minus the weight of the respective probe.

The most interesting variables in the hierarchical Bayesian graphical model are on the group level because they allow us to compare the difference between the salience and neutral condition. The relation between the weight for *T*_probe_ in the salience condition ω_sp_ and its counterpart in the neutral condition ω_np_ shows if salience has an influence on attention parameters (see Figure 5). The parameter distribution for the weights are depicted in Figure 5. The parameter estimations show that *w*_sp_ = .507 and ω_np_ = .516 differ only slightly. Interestingly, the value .5 is not among the 95% of the most probable parameters for ω_np_—that is, attention is not distributed equally across the two targets in the neutral condition. Because all elements were equally salient in this condition, visual properties cannot be the cause of the higher attentional weight for *T*_probe_. The temporal properties, however, offer an explanation: *T*_probe_ was always shown 150 ms after display onset. This fixed interval made it predictable. In order to measure the effect of salience unbiased by that of temporal expectation, we subtracted the deviation from the expected neutral weight .5 in the ω_np_ parameter from the ω_sp_ parameter. The corrected weight is ω_sp_ _clean_ = .493. The correction shifts the weight of the salience condition ω_sp_ in the opposite of the expected direction, which would be an increased weight for the salient stimulus. As explained earlier, the effect is small and hence again, ω_np_ and ω_sp_ _clean_ differed only slightly.

**Figure 6.**
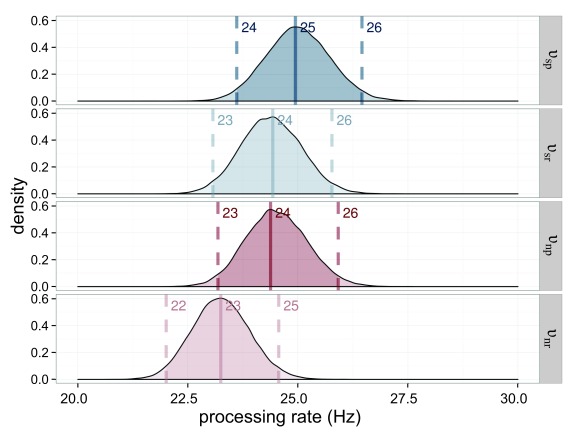
Estimated processing rates (υ) for Experiment 1. The processing rates of the salience condition (υ_sp_ = processing rate for the salient probe; υ_sr_ = processing rate for the reference in the salient probe displays) are shown in blue, those of the neutral condition (υ_np_ = processing rate for the neutral probe; υ_nr_ = processing rate for the reference in the neutral probe displays) in red. The darker distributions belong to the probe stimulus and the lighter distributions belong to the reference stimulus.

The processing rates for the stimuli are very similar. All are in the range of 23.3 Hz to 24.9 Hz. This result is to be expected when both weights and capacities are similar (see Figure 6).

**Figure 7.**
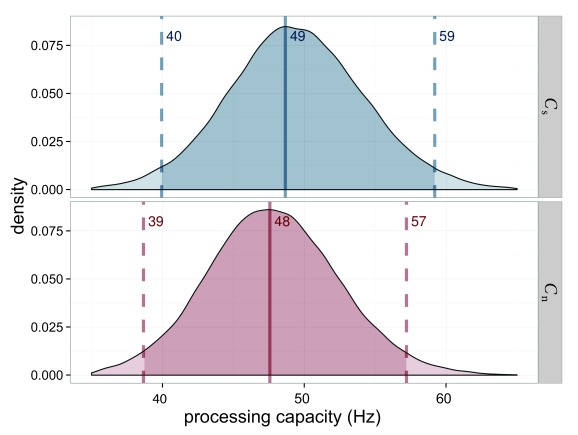
Estimated processing capacities (*C*) for Experiment 1 in the salience condition (*C*_s_ = capacity for salient stimulus) in blue and the neutral condition (*C*_n_ = capacity for neutral stimulus) in red. The difference of 0 is in the highest density interval (HDI) if both distributions are subtracted, which indicates that the overall processing capacity was similar in both conditions.

The processing capacity was similar in both conditions with *C*_s_ = 49.4 Hz and *C*_n_ = 48.1 Hz (see Figure 7). The distribution of its difference is centered on 0. Hence a difference is very unlikely. Importantly, this allows one to compare the attentional weights across conditions because it can be assumed that the same process distributes the same resources differently in the two conditions.

The posterior predictive (Figure 4) serves two purposes: It is a plausibility check of the model and compresses the evidence for the parameters in a prediction for new data. Because the parameters are given as distributions, the certainty of the predicted data can be indicated by the color gradient in the figure. For the current experiment, the conditions are strongly overlapping—that is, salience does not affect processing speed or attentional weights, and consequently the judgments are similar in both conditions.

### Discussion

Staying close in design to the well-established TOJ paradigm while using multi-stimulus displays yielded plausible data that resembled psychometric functions. The TVA-based model was successfully applied to model the data. It was possible to estimate parameter distributions for individual participants as well as on the group level. These rates are comparable to what has been found in earlier TVA studies (e.g., Finke et al., 2005). In sum, this allows us to use TOJs on multi-element displays in order to compute TVA-based attentional parameters.

Although one stimulus was clearly salient due to its 90° orientation difference, this salience did not increase its attentional weight nor its processing rate in comparison to its counterpart from the neutral condition. Salience thus had no influence on the distribution of attention as measured by TVA parameters. This result cannot be attributed to a lack of sensitivity: The fact that the neutral weight (.5) was located outside of the HDI for the neutral condition (likely due to the fixed time of the *T*_probe_ onset) indicates the sensitivity of the approach. That is, if present, even small differences between attentional parameters of *T*_reference_ and *T*_probe_ should have been detected.

The absence of a salience effect on attentional parameters might be explained by the lack of a delay between the property which is supposed to guide attention (the local contrast) and the events which are relevant for the TOJ—that is, the onsets. TVA assumes that the sensory evidence for onset and local contrast is available equally fast. In the V1-salience model by Li (2002), however, it is assumed that salience is computed by pyramidal cells and interneurons that interact locally and reciprocally in their layer. The onset, however, can be processed by a simple feed-forward network (VanRullen & Koch, 2003). If the sensory evidence for salience is indeed not available fast enough, this would explain why the attentional weights are unaffected by salience. This explanation also fits the results of Dombrowe et al. (2010) on the time course of salience.

The following experiments changed the temporal feature of the targets. The events to be judged are target offsets in Experiment 2 and brief flickers in Experiment 3.

## Experiment 2

In Experiment 2, the onsets used in Experiment 1 were replaced with offsets. Offsets are susceptible to attentional effects (Vingilis-Jaremko, Ferber, & Pratt, 2008). We hypothesized that the presence of the salience-generating property prior to the event (offset) should cue the event and hence lead to a higher attentional weight. Again, this should lead to a quicker encoding into VSTM. The offset at the potentially salient position occurred 150 ms after the onset of the display. As shown by Donk and Soesman (2010), effects of orientation salience should be present in this time range.

### Method

#### Participants

A total of 20 participants (9 male and 11 female; *M*_age_ = 22.6, range 19-47), including the authors, participated in Experiment 2. All of them were students or members of Leuphana University of Lüneburg or Paderborn University. Each participant reported normal or corrected-to-normal visual acuity and completed one session. All participants except for the authors received a payment of 8 Euro per hour.

#### Apparatus

The apparatus was the same as in Experiment 1.

#### Stimuli

The same stimuli as in Experiment 1 were used. Because this time the temporal order of offsets had to be judged, all elements (background elements, *T*_reference_ and *T*_probe_) were shown after the initial presentation of the fixation cross. The offsets of the two targets occurred with the same timing as the onsets in Experiment 1.

#### Procedure

The procedure was the same as in Experiment 1 except that participants were instructed to judge which element disappeared first. This is depicted in Figure 3.

### Results

**Figure 8.**
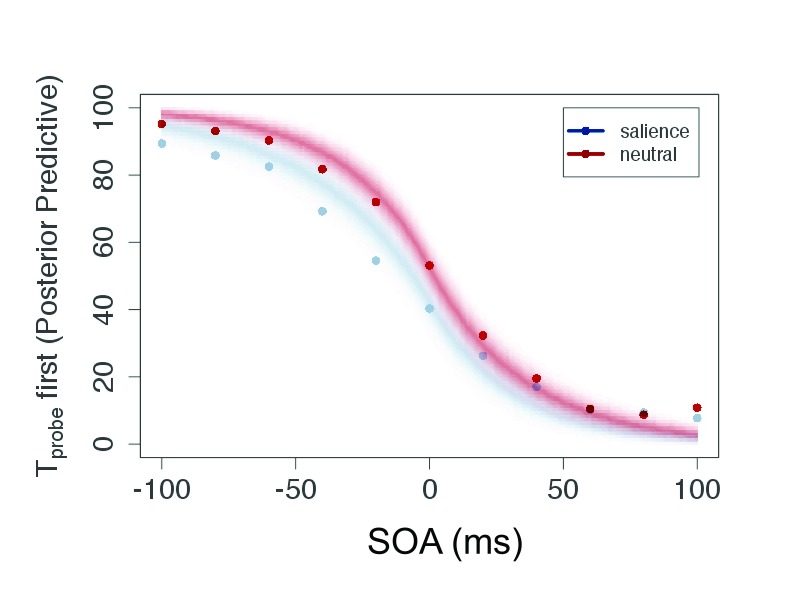
Plot of raw data (mean of judgment frequency per SOA over all participants) and posterior predictive for the salient and neutral condition of Experiment 2. This plot shows predicted data based on the estimated parameters. SOA = Stimulus Onset Asynchrony.

Similar to Experiment 1, the data resembled psychometric functions. Hence, it was possible to apply the model and estimate the parameters. A summary of the raw data is given in Figure 8.

**Figure 9.**
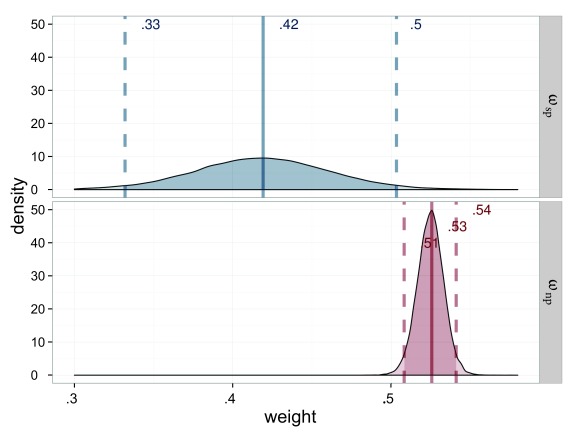
Estimated attentional weights (ω) for the probe stimuli of Experiment 2, salience condition (ω_sp_ = weight for the salient probe) in blue and neutral (ω_np_ = weight for the neutral probe) in red. The weights for the reference stimuli are 1 minus the weight of the respective probe.

**Figure 10.**
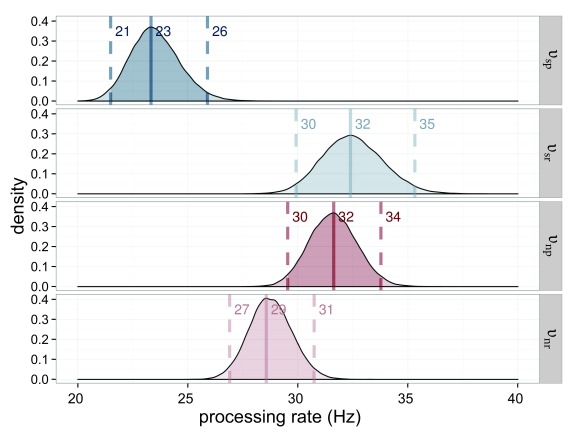
Estimated processing rates (υ) for Experiment 2. The processing rates of the salience condition (υ_sp_ = processing rate for salient probe; υ_sr_ = processing rate for reference in salience displays) are shown in blue, those of the neutral condition (υ_np_ = processing rate for neutral probe; υ_nr_ = processing rate for reference in neutral displays) in red. The darker distributions belong to the probe stimulus and the lighter distributions belong to the reference stimulus.

**Figure 11.**
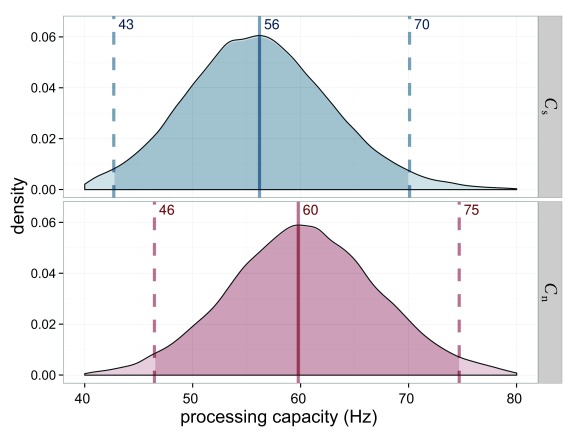
Estimated processing capacities (*C*) for Experiment 2 in the salience condition (*C*_s_, in blue) and the neutral condition (*C*_s_, in red). The difference of 0 is in the highest density interval (HDI) if both distributions are subtracted, which indicates that the overall processing capacity was similar in both conditions.

The attentional weights on the group level are, again, most informative about whether attention was deployed unequally. In contrast to Experiment 1, the attentional weight for the probe in the salience condition, ω_sp_ _clean_ = .393, clearly differed from the equal weight distribution, as shown in Figure 9. As in Experiment 1, the attentional weight ω_np_ = .526 in the neutral condition deviated from the balanced value of.5. We suppose this deviation to be a consequence of the timing which differed for probe and reference stimulus. The weight in the salience condition was again corrected (uncorrected ω_sp_ = .423), such that the small shift in weight likely due to timing does not affect the measurement of salience. The processing rate for the salient υ_sp_ = 23.4 Hz was lower than the processing speed for the neutral condition υ_np_ = 31.6 Hz (see Figure 10 for their distributions). The processing capacity, as shown in Figure 11, was constant over the conditions which allowed the comparison of weights across conditions. The comparison of the judgment data and the posterior predictive in Figure 8 shows that the model is able to fit the data and provides a reasonable description for them.

### Discussion

Replacing the onset from Experiment 1 with the offset led to a distinct and measurable salience effect. The attentional weights shifted away from the salient to the non-salient stimulus. Contrary to theory, the salient stimulus received less attentional weight and hence was processed slower than the non-salient target which in turn means that the offset of the salient target raced slower to VSTM.

This finding is not in line with the results by Vingilis-Jaremko et al. (2008), which originally motivated the use of offset events. Other findings on time perception and from designs based on response time, however, are better compatible with the results of Experiment 2. For example, New and Scholl (2009) reported that the subjective duration of an attended stimulus is longer than the duration of an unattended one which contributes to a delayed perceived offset of the attended stimulus. Similarly, Rolke, Ulrich, and Bausenhart (2006) showed that the response to a cued offset takes longer than the response to an uncued offset. They conclude that attention delays the perceived stimulus offset. Furthermore, the absence of a stimulus can be salient if it violates the local pattern (Li, 2002). Replacing the stimuli with gaps might hence have caused an unwanted manipulation of salience. Although we cannot offer a full explanation yet, it is likely that the unexpected direction of the effect caused by salience is due to the offset event. This event does not only probe salience but also manipulates it. Independent from this unexpected finding, Experiment 2 however substantiated the validity of the method proposed in the present paper. The TVA-based analysis was applicable to the data and yielded interpretable parameters.

Experiment 3 makes a final attempt at disclosing effects of salience with this method by keeping the salience display as constant as possible.

## Experiment 3

Although a salience effect was measured successfully in Experiment 2, its direction was unexpected. We hypothesized that the offset event was responsible for this because it changed the salience display permanently. Therefore, a short flicker was used in Experiment 3. The flicker prevents a permanent change of the salience display. Again, salience is supposed to increase the attentional weight and thus speed up the processing of the probe stimulus.

### Method

#### Participants

A total of 19 persons (2 male and 17 female; *M*_age_ = 22.0, range 19-28) participated in Experiment 3. All of them were students at Leuphana University of Lüneburg. Each participant completed one session, reported normal or corrected-to-normal visual acuity and again received a payment of 8 Euro per hour.

#### Apparatus

The apparatus was the same as in Experiment 1.

#### Stimuli

The same stimuli as in Experiment 1 were used. To avoid the effects of both onset and offset, the targets flickered. The flicker was realized by removing each target for 80 ms. The timing was otherwise similar to Experiments 1 and 2.

#### Procedure

The procedure was the same as in Experiment 1 except that the participants were instructed to judge whether the first flicker was on the left or the right of the fixation cross. This procedure is depicted in Figure 3.

### Results

**Figure 12.**
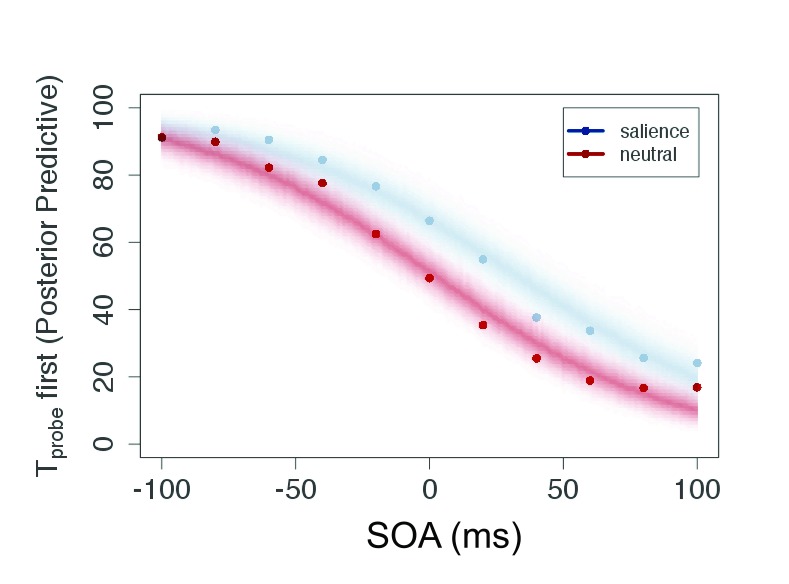
Plot of raw data (mean of judgment frequency per SOA over all participants) and posterior predictive for the salient and neutral condition of Experiment 3. This plot shows predicted data based on the estimated parameters. SOA = Stimulus Onset Asynchrony.

As in the previous experiments, it was possible to apply the model to the TOJ data and to derive the parameters. For illustration, the averaged responses per SOA are given in Figure 12.

**Figure 13.**
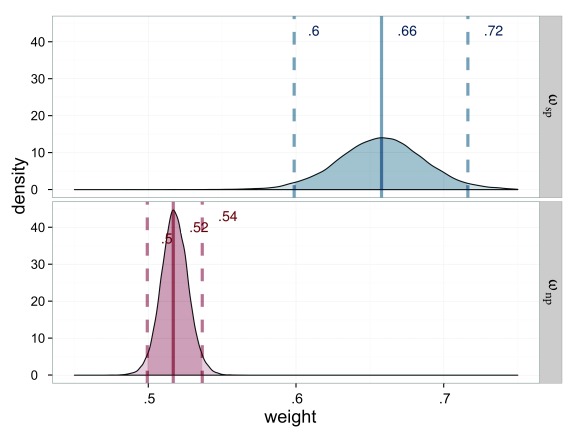
Estimated attentional weights (ω) for the probe stimuli of Experiment 3, salience condition (ω_sp_) in blue and neutral condition (ω_np_) in red. The weights for the reference stimuli are 1 minus the weight of the respective probe.

As already suggested by the different trends in the figure, the attentional weights show a clear and distinct advantage for the salient *T*_probe_ which is ω_sp_ _clean_ = .643 in comparison to the non-salient target ω_np_ = .518. We again found a small attentional effect due to the fixed interval between onset of the display and the event occurring at the *T*_probe_ stimulus and corrected for it as explained in the results of Experiment 1 (uncorrected ω_sp_ = .658). The estimated weight distributions are shown in Figure 13.

**Figure 14.**
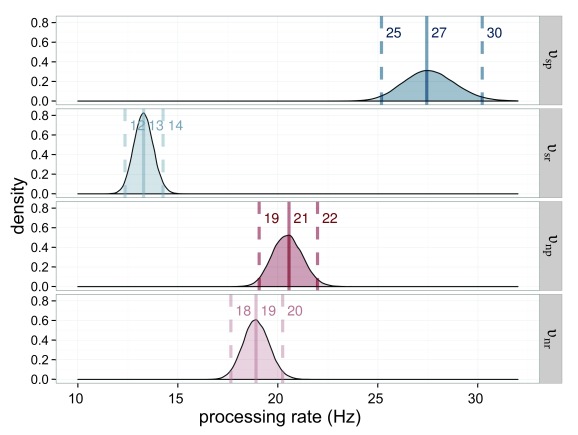
Estimated processing rates (υ) for Experiment 3. The processing rates of the salience condition (υ_sp_ = rate for the salient probe; υ_sr_ = rate for the reference in the salient display) are shown in blue, those of the neutral condition (υ_np_ = rate for the neutral probe; υ_nr_ = rate for the reference in the neutral probe display) in red. The darker distributions belong to the probe stimulus and the lighter distributions belong to the reference stimulus.

This result also means that processing speed changed: The salient element is processed faster υ_sp_ = 27.5 Hz than its non-salient counterpart from the neutral condition υ_np_ = 20.6 Hz while the reference stimulus from the salience condition is processed slower υ_sr_ = 13.2 Hz than its counterpart υ_nr_ = 18.09 Hz. All estimated rate parameters are shown in Figure 14. The rates can be interpreted as a shift of resources from the non-salient reference stimulus to the salient probe stimulus in the salience condition.

**Figure 15.**
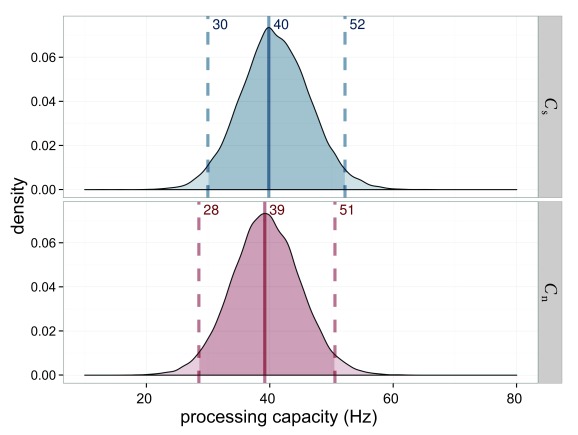
Estimated processing capacities (*C*) for Experiment 3 in the salience condition (*C*_s_; blue) and the neutral condition (*C*_n_; red). The difference of 0 is in the highest density interval (HDI) if both distributions are subtracted, which indicates that the overall processing capacity was similar in both conditions.

The overall processing capacity, again, was the same in both conditions as shown in Figure 15. Hence, weights are interpretable as a redistribution of the same resources.

Also and as the final result of modeling, the posterior predictive shows a distinct shift between the salient and neutral condition as depicted in Figure 12. The two conditions show almost no overlap. This shift indicates that the salient *T*_probe_ is perceived earlier, in perfect accord with the parameters and summary of the raw data discussed above.

### Discussion

Experiment 3 yielded a salience effect that increased the attentional weight on the salient stimulus and hence its processing speed. This is in line with both the salience and TVA literature and shows that TVA can be used to quantify the effects of salience on processing. This quantification happens in terms of the individual processing speed and the attentional weight. The attentional weight describes the allocation of attention across all relevant stimuli and has the advantages of measuring the salience in relation to the other stimulus in the display. Attentional weights are directly comparable if overall capacity is the same. The processing speed is a second possible measure of salience. Though attentional weight is theoretically more sound, processing speed is directly comparable even if the capacity does not stay the same.

With a value of ω_sp_ _clean_ = .642, the shift from the neutral weight of .5 is very clear. Note that the TOJ method is rather conservative in this respect because both targets have to be encoded. This makes extreme values for the attentional weight close to 0 or 1 very unlikely.

To the best of our knowledge, this is the first study in which TOJs manipulated by salience were sufficiently sampled to show the full psychometric function and the occurrence of systematic shifts in the report probability. The occurrence of this shift was already assumed by Donk and Soesman (2011). Because only one-half of the suspected psychometric function was sampled in their experiment, the actual function was not derivable. Both the data presented in Figure 12 and the posterior predictive show the expected shift in the function which speeded processing of a flickering salient element compared to a flickering non-salient element. This experiment shows that salience can lead to prior entry as already reasoned by Donk and Soesman.

The size of the change in attentional weights (as inferred from the HDI and the posterior predictive) indicates that the proposed method will be appropriate to prove effects of different size, including small effects: There is nearly no overlap between the expected psychometric functions for the salient and neutral condition. This means that smaller shifts will also be detectable, as, for instance, can be expected when smaller local differences would be used. The small but reliable effect of fixed time of the *T*_probe_ shows that the method is sensitive enough for small effects.

The arguments why to prefer the TVA model over the classical analysis by psychometric functions are theoretical ones, as explained in the Introduction. We, however, also conducted a conventional analysis of psychometric functions which the interested reader finds in the Appendix. It is in accord with the present results but provides less information.

## Experiment 4

Experiment 3 showed the feasibility of the proposed method. Experiment 4 was designed as a test of the generality of our approach. We furthermore analyzed feature differences smaller than the admittedly large difference between 0° and 90° in Experiments 1 to 3. To this end, we used a high-salience condition and a low-salience condition, operationalized by stimulus luminance.

### Method

#### Participants

A total of 30 persons (14 male and 16 female; *M*_age_ = 25.7, range 19-48), including all authors, participated in Experiment 4. All were students or members of Paderborn University. Each participant completed one session, reported normal or corrected-to-normal visual acuity and again received a payment of 8 Euro per hour (except for the authors).

#### Apparatus

The apparatus was the same as in Experiment 1.

#### Stimuli

The same stimuli as in Experiment 1 were used, except that salience was manipulated in the luminance dimension. In the low-salience condition, a dark gray probe with RGB (80, 80, 80) (4.03 cd/m^2^) was used. In the high-salience condition, the probe was black RGB (0, 0, 0) (0.31 cd/m^2^). To keep the experiment as short as possible, the neutral condition without a salient probe was omitted. We did this because Experiment 3 already showed what can be theoretically assumed: This condition yields a weight of .5 for the target—that is, attention is distributed equally between the two visually equal targets.

#### Procedure

The procedure was the same as in Experiment 3.

### Results

**Figure 16.**
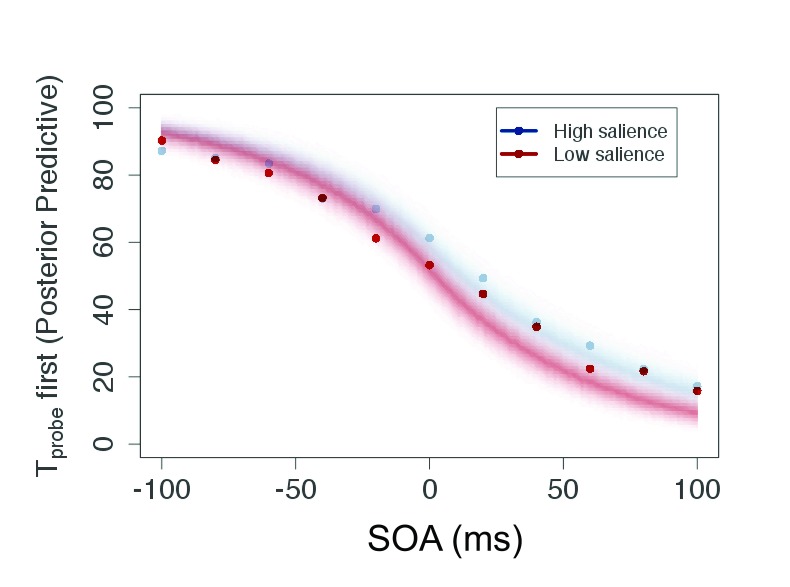
Plot of raw data (mean of judgment frequency per SOA over all participants) and posterior predictive for the high-salience and low-salience condition of Experiment 4. This plot shows predicted data based on the estimated parameters. SOA = Stimulus Onset Asynchrony.

**Figure 17.**
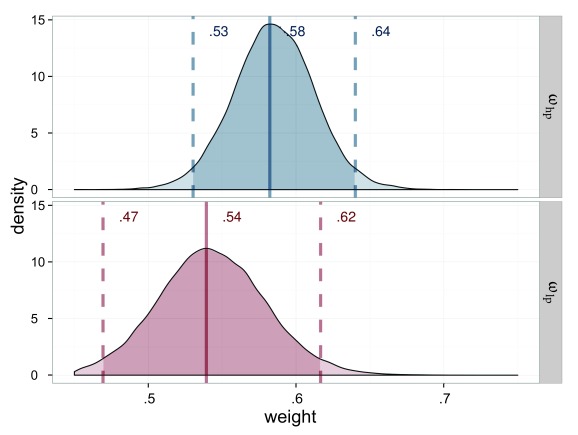
Estimated attentional weights (ω) for the probe stimuli of Experiment 4, high-salience condition (ω_hp_) in blue and low-salience condition (ω_lp_) in red. The weights for the reference stimuli are 1 minus the weight of the respective probe.

Again, the raw data were typical TOJ data (see Figure 16). The attentional weight for the probe in the high-salience condition ω_hp_ = .582 was higher than in the low-salience condition ω_lp_ = .539, which implies a difference of .043 in attentional weight. The parameter distributions are shown in Figure 17.

**Figure 18.**
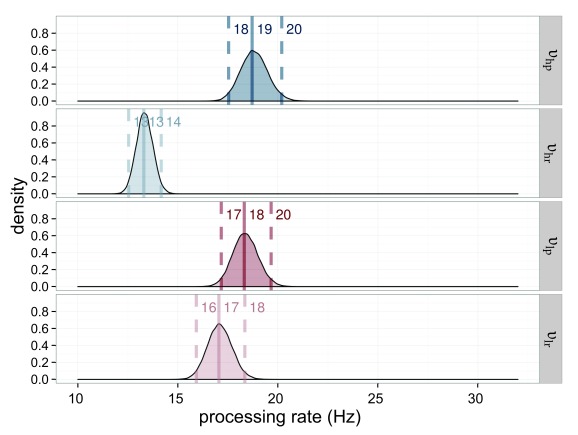
Estimated processing rates (υ) for Experiment 4. The processing rates of the high-salience condition (υ_hp_ = rate for the highly salient probe; υ_hr_ = rate for the reference in the high-salient probe displays) are shown in blue, those of the low-salience condition (υ_lp_ = rate for the lowly salient probe; υ_lr_ = rate for the reference in the low-salient probe displays) in red. The darker distributions belong to the probe stimulus and the lighter distributions belong to the reference stimulus.

Figure 18 depicts the processing rates. This figure shows that the difference between the high- and low-salience condition lies mainly in the processing of the non-salient reference stimulus: High- and low-salience probes were processed nearly equally fast with a rate of υ_hp_ = 18.7 and υ_lp_ = 18.3. The processing speed of the reference stimulus, however, varied strongly with condition, with a rate of υ_hr_ = 13.3 in the high-salience and one of υ_lr_ = 17.1 in the low-salience condition. This is important for the theoretical explanation (see below).

**Figure 19.**
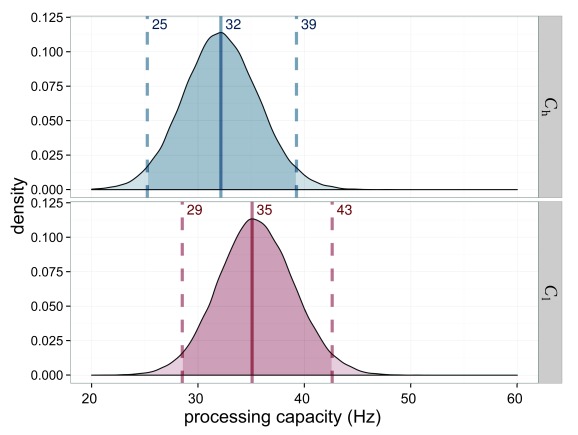
Estimated processing capacities (*C*) for Experiment 4 in the high-salience condition (*C*_h_; blue) and the low-salience condition (*C*l; _h_). The difference of 0 is in the highest density interval (HDI) if both distributions are subtracted, which indicates that the overall processing capacity was similar in both conditions.

The overall processing capacity was very similar with *C*_h_ = 32.2 for the high-salience condition and *C*_l_ = 35.1 for the low-salience condition, as depicted in Figure 19.

The posterior predictive, presented in Figure 16, shows an asymmetrical distribution. This accords to the processing speeds shown in Figure 18: The processing of the reference targets is affected more than the processing of the probes.

### Discussion

Experiment 4 expanded the scope of the present method to the luminance dimension and tested two quantitative levels of salience. As expected, both singletons received increased attentional weight, and this increase scaled with their salience: The highly salient probe received more attentional weight than the less salient probe. Thus, this fourth experiment shows that the proposed method is applicable to features other than orientation, which is a promising result for further generalization. Furthermore, Experiment 4 indicated that quantitative differences in salience lead to quantitative differences in attentional weights. This result promises to enlarge the scope of our method to a general quantitative model of salience. Note, however, that this difference seems to be caused by slower processing of the reference stimuli. Faster processing of the highly salient compared to the less salient probe contributed only slightly to this difference.

## General Discussion

The Theory of Visual Attention (TVA) can serve as a foundation for quantifying visual salience. We showed this by conducting four experiments. All experiments substantiate the soundness of the model, which combines TOJs and TVA.

Experiment 1 demonstrated the applicability of the suggested method in general. This was achieved by combining salience displays and TOJs. Experiment 2 tested the effects of salience on attentional weights and processing speed. Although in principle successful—the experiment indeed measured effects on weight and speed—it was not entirely satisfying because attentional weights favored the non-salient stimulus, which was processed faster than the salient one.

As we reasoned that the offsets we used in Experiment 2 might not have been optimal because they caused (possibly) salient gaps in the bar array, we replicated the experiment with flickering stimuli. This experiment showed the full relevant data pattern: The salient stimulus received more attentional weight and was processed faster than the non-salient one. Attention was withdrawn from the non-salient stimulus and redistributed to the salient one.

In Experiment 4, we applied the flicker procedure to the luminance dimension in order to demonstrate its applicability to other stimulus dimensions as well as its sensitivity and its usefulness for a quantification of salience effects. All aims were successfully reached by Experiment 4: Salient stimuli drew attention towards themselves, and there was a difference in weights and processing speeds between highly and less salient stimuli.

Beyond comparison of individual model parameters, both experiments have shown that salience redistributes resources according to feature differences. Attention which is dedicated to the salient stimulus is withdrawn from the reference stimulus. Importantly, this relation is not predefined by the TVA model. Because the processing rate of each stimulus is modeled as an independent process, it is possible that only the salient stimulus gains while the speed of the race stays constant for the reference stimulus. (Such a rate increase would result in a capacity difference between conditions.) Although we focused on a measure of salience, this may be understood as evidence for parallel processing rather than a guided serial processing as in the Guided Search models by Wolfe (e.g., 1994, 2007) that predict an increase of attention for salient stimuli.

Independent of the salience-related results, the proposed method of combining salience displays with TOJs and TVA parametrization was successful in all four experiments: All yielded psychometric functions as well as plausible parameters including the attentional weights and processing speeds of the two targets as well as the overall processing capacity.

To sum up, the combined TVA/TOJ method proposed in the present paper seems a promising tool. Further studies could test and model the quantitative relationship between salience values and attentional weights in more detail, for instance, by employing several levels of salience instead of only two. Also, different salience dimensions could be compared directly via attentional weights, relating the salience of, say, a colored singleton to an orientation or luminance singleton. This is, however, beyond the scope of the present article.

We propose the presented procedure to measure the strength of salience because this strength can be quantitatively expressed in a theoretically meaningful parameter of a tried and tested theory. Different from earlier approaches, the method is not limited to specific salience dimensions because the task is largely independent of the type of elements. Also, it is not limited to a reference stimulus like the methods proposed by Nothdurft (2000), and Huang and Pashler (2005).

A further advantage is that no assumptions about contested issues such as the relative contribution of top-down and bottom-up influences have to be made to apply the present approach. While Theeuwes (2004, 2010, 2013), for example, takes the stance that salience captures attention inevitably, other researchers claim that all salience effects are modulated by top-down task sets (e.g., Ansorge & Becker, 2014; Folk, Remington, & Johnston, 1992; Yantis & Egeth, 1999). Our method provides a useful salience measure for both perspectives. Furthermore, interactions between bottom-up and top-down influences can be studied within the TVA framework. Nordfang et al. (2013) have developed a TVA extension that tackles this problem (see also Bundesen, Vangkilde, & Petersen, 2015). Both feature contrast and task relevance are modeled as individual variables affecting the attentional weight. That is, these authors already proposed a model for the interaction of bottom-up and top-down influences on attention. Its empirical application is, however, restricted to the partial report and the stimuli suitable for the partial report, whereas our TOJ-based approach can deal with all kinds of stimuli.

Besides effects of salience, we consistently detected a small effect on the attentional weight in the neutral conditions of Experiments 1 to 3. All visual features were equal for the two targets in these conditions, except for their timing. While *T*_reference_ varied according to the SOA, *T*_probe_ was always shown at a fixed point in time. With this procedure, the strength of salience is not distorted by the time course of salience. As a trade-off, we accepted the chance that an effect of predictability occurred—which indeed was the case. The formal model, however, allowed to correct for it. Note that this finding is well in line with results from Vangkilde, Coull, and Bundesen (2012), who investigated the effect of temporal predictability on perception. They examined effects of timing on t0, the minimal effective exposure duration, and the processing speed υ, whereas we detected an influence on the attentional weight ω of the predictable stimulus and its υ parameter. The precision with which the small effect was detected is promising for future studies.

A further aspect concerning the timing of the experiment is the presentation duration of the display prior to the TOJ. Although we kept it equal in all conditions, decreasing and increasing the duration of the salience display is possible. By this procedure, effects of presentation duration—as in research on the time course of salience—can be related to attentional weight. Note however that the TOJ might not be optimal for this because it requires a minimal time range for the two stimuli to be presented.

Besides the advantages of theory and Bayesian analysis, there are also drawbacks to the method proposed in the present paper. A weak point is that a TOJ requires a temporal event that can stand out against the salience manipulation without overriding it. For the attentional weight advantage of salient stimuli, the type of change did matter. Salience has, as demonstrated by Experiment 1, next to no influence on the attentional weight when onsets are detected. The precision of the approach can, however, be used to further investigate the reason for this finding, for example, to test whether onset information is available before salience information.

To sum up, the metrics of TVA allow a precise, general, and sensitive quantification of the effects of salience. This metric can be measured in plausible parameters backed by theory. Different from earlier approaches, the present method is not limited to specific stimuli, and presentation duration can be controlled well to take the time course of salience into account. Given the sensitivity of the method, it is likely that gradual changes of local differences can be tested. Also, the method allows combining salience from different dimensions and thus offers the possibility to examine whether salience effects exhibit an underlying general metric. That is, the approach discussed in the present paper might offer a new method of quantifying visual salience.

## Appendix A Bayesian Parameter Estimation for the Proposed Model

We use Bayesian statistics for the formalization of the model and data analysis because Bayesian methods are particularly well-suited for inference under an assumed model (Little, 2006). We thus implemented a generative model based on the mathematical description of TVA to perform Gibbs sampling (Plummer, 2003) to obtain posterior distributions of all relevant parameters and comparisons.

We employed the hierarchical Bayesian graphical model as shown in Figure 2 for analyzing the data. The discrete y nodes correspond to the collected data and represent the response for *T*_probe_ as the first target. The *n* node represents the total amount of trials per SOA. θ represents the probability of a success of the binomial distribution at each SOA. This probability and the TVA parameters introduced in the TVA section are computed according to the equations in Table 1. This describes the model for a neutral condition, in which no stimulus is salient. The salience condition, in which one of the stimuli was salient, was modeled analogously.

Besides the data, priors are a mandatory part of Bayesian data analysis. Usually, these priors express previous knowledge about a variable. To keep the priors and resulting analysis unbiased by assumptions and previous data (which the reader might not agree with) we based the priors on the theoretical extrema of these parameters. This type of prior is called non-informative. The priors of weight and processing capacity are bound by 0 on the lower end. Also, the weight cannot exceed 1. The processing capacity is not limited explicitly by theory, but the chosen maximum processing capacity of the prior (500 Hz) would imply that a race to VSTM could be completed in 2 ms which is highly unlikely. As recommended by Gelman (2006) we decided for a *t*-half-distribution, the positive half of a *t*-distribution, for the variance priors of our hierarchical model. The priors are given in Table A1.

**Table A1.** Prior Distributions

*w*_npm_∼dunif(0,1)
*w*_npτ_dt(0,1,1)*T*(0,)
*C*_nm_∼dunif(0,500)
*C*_nτ_∼dt(0,1,1)*T*(0,)

*Note*. The index *m* denotes the mean and the index τ denotes the precision for the processing capacity *C*_n_ and the attentional weight ω_np_.

Given the model, priors and collected data, the parameters are computed by Gibbs Sampling (Plummer, 2003). Mathematically, it is guaranteed that for an infinitely long process the result is perfectly representative of the true posterior. Limited by computing time and resources, the process is, however, not guaranteed to converge. Hence, it is crucial for a Bayes analysis to check the samples for representativeness. The parameters which have been used for Gibbs Sampling are a burn-in period of 5,000 iterations, 100,000 samples and a thinning of 10 in four chains. As explained by Kruschke (2010, p. 131), there is no optimal way of checking representativeness but rather several ways that are useful. The four chains converged for all reported posterior distributions. This was checked by examining the trace plots, the histograms (density plots), and the shrink factor. Additionally, the accuracy of the samples was checked by the effective sample size. All these parameters indicated that our chains indeed converged.
